# Cold Dash in the Asphyxia of Infants

**Published:** 1842-05

**Authors:** 


COLD DASH IN THE ASPHYXIA OF INFANTS.

Dr. Sherer, from whose letter we have just quoted, has on the same sheet furnished us with the following fact:—

"A negro woman whom I lately attended, had a pelvis so narrow in proportion to the size of the head of the fœtus, that the latter was a long time in passing the straits, and when delivered was elongated to an extraordinary degree. Respiration did not commence, and all the usual means were employed without effect, except that a slight muscular contraction was once observed. At this moment one of the attendants (all of whom were of the Catholic faith), performed the baptismal rite; and in the hurry of the occasion the water was very profusely applied to the head and breast of the child. In less than half a minute it began to cry, and has ever since remained healthy."

For many years we have been in the habit of applying cold water, suddenly and with force, to the chests of new-born infants, when they were perfectly or partially asphyxiated, and regard it as superior to every other meaus of resuscitation. D.

